# Tibetan Medicines for the Treatment of Diabetic Nephropathy

**DOI:** 10.1155/2021/7845848

**Published:** 2021-10-06

**Authors:** Lili Pu, Chunhong Yang, Liqiong Yu, Shiling Li, Yaqin Liu, Xinan Liu, Xianrong Lai

**Affiliations:** ^1^School of Pharmacy, Chengdu University of Traditional Chinese Medicine, Chengdu 611137, China; ^2^School of Ethnic Medicine, Chengdu University of Traditional Chinese Medicine, Chengdu 611137, China

## Abstract

As an important part of the traditional Chinese medicine system, Tibetan medicine has its unique treatment methods for diabetes mellitus and its complications. Diabetic nephropathy (DN) is one of the most serious diabetic microvascular diseases. Tibetan medicine believes that the occurrence of DN is closely related to renal function changes, and it can be effectively prevented and treated by improving renal lesions. In this paper, we consult ancient books of Tibetan medicine and summarize the medicines that treat kidney disease in the Tibetan medicine system. The Chinese name, English name, and Latin name of these drugs were searched as keywords in the online database. Thirty-four drugs were found for the treatment of DN. The most commonly used were *Amomum kravanh*, *Terminalia chebula*, and *Tribulus terrestris*, and we introduced the traditional uses and modern pharmacological activities of these drugs. The results indicate that Tibetan medicines for kidney disease could be used as potential candidate drugs for DN; they would expand the range of medications for DN and provide a new idea for the treatment of DN.

## 1. Introduction

Diabetic nephropathy (DN) is a series of microvascular complications caused by changes in renal structure and function due to chronic diabetic microangiopathy. It is mainly characterized by continuous albuminuria and progressive reductions in renal function. It is one of the most serious chronic complications of diabetes mellitus and one of the most common causes of end-stage renal disease (ESRD) [1]. If it is not treated promptly, it will endanger the life and health of patients. According to relevant epidemiological statistics, diabetic patients are expected to reach 578 million in the world by 2030; meanwhile, DN patients will break through 100 million [2]. The incidence of DN in diabetic patients in China is about 47.66% [3]. Its pathological features include early glomerular hyperfiltration, changes in capillary permeability, increase in mesangial membrane cells and matrix, glomerular basement membrane thickening, deposition of extracellular matrix, subsequent glomerular sclerosis and fibrosis, and ultimately leading to the deterioration of renal function [4], which is the main cause of death in diabetic patients. The pathogenesis of DN is complex. Modern medicine mainly prevents and treats DN by controlling blood glucose and blood pressure, regulating blood lipids, lowering urinary albumin, and following other principles. But diabetes mellitus and its complications cannot be treated well by these simple means only [5, 6]. So it is necessary to combine modern medical treatments with traditional medical methods to obtain better therapeutic results.

Tibetan medicine is an important part of the treasure house of Chinese medicine, which has a long history, complete theory, and rich content. It has made great contributions to the reproduction, survival, and development of Tibetan people and has a unique curative effect on the treatment of diabetes mellitus and its complications. Based on the knowledge of DN from the Tibetan medicine theory, we have found that the occurrence of DN is closely related to kidney lesions. And modern studies have also found that DN can be effectively prevented and treated by protecting kidneys and improving kidney function [7, 8]. Tibetan medicine has a unique therapeutic approach to diabetes mellitus and its complications and has been gradually applied in the treatment of DN. However, the current records on Tibetan medicines for the treatment of DN are not comprehensive enough and lack systematic summary and generalization. Therefore, supported by Tibetan medicine theory, we attempt to find Tibetan medicines for DN from the Tibetan medicines for treating kidney diseases, in order to expand the drug use range of DN and provide new ideas for the treatment of DN.

## 2. The Knowledge of Tibetan Medicines on DN

Tibetan medicine believes that there are three major factors: rLung, Bad-kan, and mKhris-pa in the physiological activities of the human body. The three factors control the movement changes of the seven material bases (seven essences) in diet essence, blood, meat, fat, bone, bone marrow, semen, and the motion of three excretions (three filth) in stool, urine, and sweat. Under certain conditions, the three basic factors are relatively coordinated, maintaining the balance with the seven essences and the three obscenities and keeping the normal physiological activities of the body. Once one of the three factors changes, this balance will be disrupted, causing various pathological changes that can lead to disease.

DN belongs to the category of “jingnisaku disease” (Chinese direct translation: frequent urination) in Tibetan medicine, and Tibetan medicine believes that “jingnisaku disease” is a consumptive disease, which promotes the consumption and reduction of mKhris-pa (cold), thus losing its counterbalancing effect on Bad-kan (hot) and increasing the effect of Bad-kan. The decrease in mKhris-pa and the increase in Bad-kan lead to the dysfunction of rLung, which makes the originally reduced mKhris-pa in the body or tissues decrease more and more, while the originally increased Bad-kan increases more and more, and the imbalance of the three factors is getting worse, then driving the further deterioration of DN. The “jingnisaku disease” is caused by the imbalance of rLung, Bad-kan, and mKhris-pa due to external factors such as improper diet and living. The ancient book of Tibetan medicine *Rgyud bzhi* records: “due to eating salty, sweet, cold, or heavy diet, living in a humid place for a long time, growing Bad-kan size, cannot be sublimated to the essence to be absorbed and leak into the bladder, producing body fluids and causing disease.” In the *Blue Glaze*, it is said that “long-term living in damp places and other reasons weaken the function of the kidneys, unable to differentiate the dregs and essences, directly fall into the bladder, and cause frequent urination.” This is similar to modern medicine, which believes DN is dominated by persistent proteinuria and accompanied by varying degrees of frequent urination, acute pain, and urinary discomfort. Tibetan medicine believes that “jingnisaku disease” can be caused by factors such as weakened kidney function. The prescriptions for the treatment of “jingnisaku disease,” such as Shibawei Hezi diuretic pill and Shiliu Rilun pill, have the effect of benefiting the kidneys. And *Tribulus terrestris*, a Tibetan medicine for the treatment of kidney disease, has also been used in the Tibetan formula Siwei Jianghuang decoction powder for the treatment of “jingnisaku disease.” In conclusion, the occurrence of “jingnisaku disease” is closely related to kidney pathology.

## 3. Materials and Methods [9]

We have manually searched eleven Tibetan medicine monographs and drug standards, such as *Dictionary of Chinese Ethnic Medicine*, *Jing Zhu Materia Medica*, *Chinese Tibetan Materia Medica*, *Rgyud bzhi*, and *Blue Glaze*. And we have looked up the information on Tibetan medicines for the treatment of kidney diseases and used their dialect, English, or Latin names as keywords to search in Chinese online databases (such as CNKI, VIP, and Wanfang) and English online databases (such as Sci-Hub, ScienceDirect, GeenMedical, etc.) to obtain their modern pharmacological studies on DN. In addition, we consulted the Tibetan prescriptions for the treatment of renal diseases in *Treasure House of Tibetan Medicine Prescriptions* and *Interpretation of Commonly Used Tibetan Medicines* and summarized the commonly used Tibetan medicines that are used more frequently and can treat DN from these formulas.

## 4. Results

**Figure 1:**
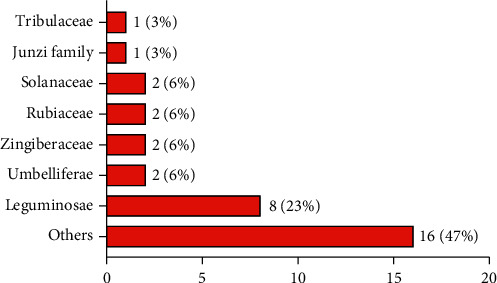
The most common families of Tibetan medicines in the treatment of DN.

In this paper, we reviewed 362 Tibetan medicines for the treatment of kidney diseases (nephritis, kidney deficiency, kidney cold, kidney heat, kidney edema, etc.) in the traditional Tibetan medicine system. Among these 362 Tibetan medicines, 60 have been used to treat kidney diseases in modern studies, of which 34 have been used for the treatment of DN. The 34 Tibetan medicines for DN are all botanicals, distributed in 23 different families, and the most common families are Leguminosae (23%), Umbelliferae (6%), Zingiberaceae (6%), Rubiaceae (6%), and Solanaceae (6%) (Figure 1). The scientific names, Chinese names, Tibetan names, families, medicinal parts, and modern pharmacological effects of the Tibetan medicines for DN are shown in Table 1. In addition, we have found that 16 Tibetan medicines for the treatment of kidney diseases have antidiabetic activity. Therefore, it is necessary to further study the effects of these Tibetan medicines on DN, with a view to using them in the treatment of DN.

### 4.1. Tibetan Medicines for Treating DN

It can be seen from Table 1 that the above Tibetan medicines can protect the kidney by improving the expression of related factors (TGF, HIF-1*α*, VEGF, SOD, etc.) in renal tissue, inhibiting the damage of renal epithelial cells, alleviating renal fibrosis, scavenging free radicals, and inhibiting lipid peroxidation and other ways, and play a role in the treatment of DN.

### 4.2. High-Frequency Tibetan Medicines

**Figure 2:**
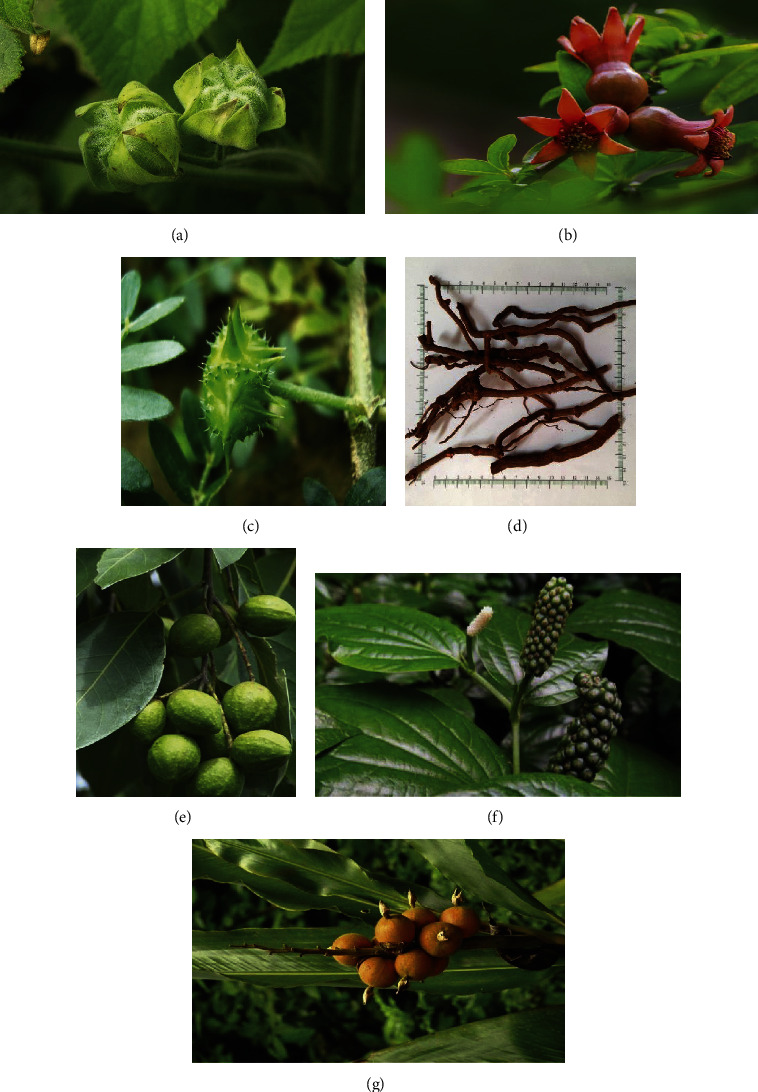
Commonly used Tibetan medicines for the treatment of DN: (a) *Malva verticillata* L., (b) *Punica granatum* L., (c) *Tribulus terrestris* L., (d) *Rubia cordifolia* L., (e) *Terminalia chebula* Retz., (f) *Piper longum* L., and (g) *Amomum kravanh* Pierre ex Gagnep.

In order to understand the frequency of Tibetan medicines use for the treatment of DN, we also inquired about Tibetan medicine prescription books such as *Treasure House of Tibetan Medicine Prescriptions* and *Interpretation of Commonly Used Tibetan Medicines* and collected 123 Tibetan medicine prescriptions for kidney disease. The Traditional Chinese Medicine Inheritance Support System (version 2.5) [74] is used to get the frequency of Tibetan medicines used in prescriptions. Through data mining, there are 7 drugs that are used more than 20 times and can treat DN (Figure 2); they are *Amomum kravanh* with the used frequency of 54, *Terminalia chebula* with 42, *Malva verticillata* with 40, *Rubia cordifolia* with 29, *T. terrestris* with 27, *Piper longum* with 25, *Punica granatum* with 22.

The following is a detailed introduction to the name base sources, traditional effects, and modern pharmacological effects of the three most widely used Tibetan medicines (*A. kravanh*, *T. chebula*, and *T. terrestris*).

#### 4.2.1. *Amomum kravanh*

This is the dried ripe fruit of the Zingiberaceae plant *A. kravanh* Pierre ex Gagnep (Tibetan name: སུག་སྨེལ། (transliterated as Jia-na-su-men) and English name: *Amomum cardamomum*). It is originated in Cambodia and Thailand and is now introduced and cultivated in Yunnan and Guangdong in China, mostly in the wet area of the trench. It is applied for the treatment of diseases such as waist and leg soreness caused by kidney cold in Tibetan clinics (*Chinese Materia Medica·Tibetan Medicine Roll*). *Blue Glaze* records that *A. cardamomum* treats cold nephropathy and various diseases caused by rLung. In *Rgyud bzhi*, *A. cardamomum* can cure kidney disease and all cold diseases.

*A. cardamomum* is widely used in the treatment of kidney diseases, and it is also recorded in Tibetan medicine. The main chemical component of *A. cardamomum* is a volatile oil, which has various pharmacological activities such as antibacterial, antioxidation, and hypoglycemic activity. *A. cardamomum* is the main medicine of Tibetan medicine compound preparations (such as Shiwei Cardamom Pill and Shibawei Hezi diuretic pill) for the treatment of kidney diseases. The modern use of cardamom has also shown some efficacy in treating many kidney diseases such as chronic renal failure, chronic pyelonephritis, and DN. Chen et al. [75] found that cardamomum volatile oil can reduce the urine protein content of acute kidney injury rats; reduce renal body index; improve renal tubular pathology; reduce MDA, NO content, and NOS activity in renal tissue; and increase SOD and GSH-Px activity in the kidney tissues so that cardamomum can improve the acute kidney injury of rats caused by gentamicin to a certain extent. Chen et al. [76] showed that *A. cardamomum* can reduce kidney damage in rats with adriamycin nephropathy, and its mechanisms may be related to the expression of TGF-*β*1 and PAI-1 in kidney tissue. They also found that *A. cardamomum* volatile oil can upregulate MMP-2 TGF-*β*1 and IGF-2 protein expression, significantly reduce the blood glucose of streptozotocin-induced DN model rats, and improve the kidney pathological changes in DN rats. In conclusion, *A. cardamomum* has a certain therapeutic effect on DN, but there are relatively few studies on its mechanisms. Therefore, modern analytical methods should be used to conduct a more extensive and in-depth experimental study on the effects and mechanisms of *A. cardamomum* in the treatment of DN, making it a common and effective drug for the treatment of DN.

#### 4.2.2. *Terminalia chebula*

This is the fruit of the Junzi family plant *T. chebula* Retz. (Tibetan name: རུ་ར (transliterated as A-ru-re)). Born in the sparse forest at an altitude of 800–1000m, mainly distributed in western and southwestern Yunnan, also cultivated in Guangdong and Guangxi. *Rgyud bzhi* records that *T. chebula* has six flavors, eight properties, and seventeen functions. Known as the “King of Tibetan Medicine” in China, it can be used in the treatment of kidney disease, diabetes, and other diseases in the Tibetan medicine system.

The effective components of *T. chebula* are complex, of which phenolic acids are the main components; it has many pharmacological activities such as antioxidation, antidiabetic, antipathogenic microorganism, anti-inflammatory, analgesic, and so on [77]. *T. chebula* has a strong antioxidant capacity, which is the basis of its efficacy in protecting kidney function and preventing diabetes, and studies have found that polyphenols are the material basis for the antioxidant effect of *T. chebula* [78]. Tayal et al. [79] found that the aqueous extract of *T. chebula* had a protective effect against oxalate-induced injury to NRK-52E and MDCK renal epithelial cells; it can enhance cell viability, reduce LDH release, and inhibit the nucleation and growth of calcium oxalate crystals, thus playing a role in protecting the kidneys. Rao and Nammi [80] found that *T. chebula* extract can reduce blood glucose in streptozotocin-induced diabetic rats, has strong antidiabetic and renal protection, and can be used for the treatment of DN. Kim et al. [81] showed that *T. chebula* extract can reduce blood glucose, blood lipids and serum MAD levels in diabetic rats caused by streptozotocin, improve the kidney pathological tissue morphology, and reduce the formation of AGEs in diabetic rats. Li et al. [11] found that *T. chebula* acid can affect the phosphorylation of VEGF-2, which cannot normally promote angiogenesis after VEGF binding to the receptor, thereby inhibiting the progression of DN. Therefore, *T. chebula* cannot only protect the kidneys and prevent and treat diabetes but also be used in the treatment of DN. It is the first choice for Tibetan medicine to treat various diseases.

#### 4.2.3. *Tribulus terrestris*

This is the mature fruit of the Tribulaceae plant *T. terrestris* L. (Tibetan name: གཟེ་མ། (transliterated as Se-ma) and English name: Tribulus). Mostly born on barren hills and fields, distributed in sandy land in Tibet and other regions. Se-ma is sweet, astringent in taste and warm in nature and functions to benefit water and dispel dampness. It is mainly used for kidney heat, urinary atresia, malnourished edema, and other diseases. *Rgyud bzhi* records that se-ma can treat diuresis and cure rheumatoid arthritis and kidney disease.

Tribulus mainly contains flavonoids, saponins, amides, and other compounds [82]; the current pharmacological research mainly focuses on *T. terrestris* saponins. *T. terrestris* saponins have pharmacological effects of antifatigue, protect myocardium, lower blood lipids, lower blood pressure, lower blood sugar, and so on [83]. Yang et al. [84] evaluated the effects of *T. terrestris* in preventing and treating renal calcium oxalate stones and found that blood urea nitrogen, creatinine, urine oxalic acid, Ca^2+^, and malondialdehyde contents of renal homogenate were increased, while blood Ca^2+^ and renal GPX content decreased significantly, renal calcium oxalate crystals was decreased, and kidney damage was alleviated after *T. terrestris* treatment. It is concluded that *T. terrestris* can effectively prevent and treat kidney calcium oxalate stones in rats and protect kidney function. Meng et al. [85] found that Tribulus can reduce the expression of LepR in the kidney, inhibit the response of the JAK2/STAT3 pathway, reduce the sensitivity of the kidney to leptin, reduce selective leptin resistance, lower blood pressure, and protect the kidney. Lamba et al. [86] found that the ethanol extract of *T. terrestris* had a significant protective effect on streptozotocin-induced diabetic rats by inhibiting oxidative stress. Zhao et al. [87] showed that *T. terrestris* extract can reduce TLR2 and TLR4 mRNA levels in pancreatic islet cells, inhibit inflammation, and significantly improve the clinical symptoms of polydipsia and polyphagia, which can be used for the treatment of type 2 diabetes. Zhao et al. [88] found that *T. terrestris* saponins also have a certain protective effect on the retina of type 2 diabetic rats. It has been reported that the hydroalcoholic extract of *T. terrestris* is able to reduce the urinary total protein and albumin content in streptozotocin-induced diabetic rats, thereby improving diabetic kidney damage and being used in the treatment of DN. In addition, among Professor Nian Li's 158 prescriptions for the treatment of DN, *T. terrestris* is the fifth most commonly used drug for the treatment of DN [89].

In summary, the high-frequency drugs used in the Tibetan medicine system for the treatment of kidney diseases are also commonly used drugs for the treatment of kidney diseases, diabetes, and DN in modern medicine. It should invest more on research to clarify its effects on DN and its mechanisms, so as to improve the safety and effectiveness of the clinical application.

## 5. Deficiencies and Prospects

Although Tibetan medicine has a positive effect on the treatment of DN, there are still many gaps and limitations in the research of Tibetan medicine. First of all, there are 362 kinds of Tibetan medicines in the Tibetan medicine system that can be used for the treatment of kidney diseases. Only 60 kinds have been proved to have kidney-related pharmacological activities; 34 kinds can be used for the treatment of DN; and most drugs still lack experimental evidence for the treatment of DN. For example, Malva seed is commonly used in the Tibetan medicine system to treat nephropathy, and it is also used by many physicians in the treatment of DN. However, there is no research on its biologically active ingredients and pharmacological mechanisms in the treatment of DN. Therefore, it is necessary to carry out deep researches combined with modern medical research methods to clarify the bioactive components of these drugs and their action and mechanism on DN, so as to be used in the clinical treatment of DN as soon as possible. Secondly, we found that among the 60 kinds of Tibetan drugs for treating kidney diseases, 16 have antidiabetic activity, but there is no research report on DN. For example, modern research has found that *Althaea rosea* can be used for the treatment of kidney stones, kidney damage, and so on. The hypoglycemic and antioxidant capacity of its seeds indicate that it has a certain effect in the treatment of diabetes and its complications [90, 91], but there is no experimental research on DN at present. Therefore, we hope to invest some researches on these drugs to determine their effects on DN and find more drugs for the treatment of DN. In addition, the potential toxicity of Tibetan medicines should not be overlooked. For example, Radix Rubiae has a good therapeutic effect on DN, but some studies have found that the alcohol extract of Radix Rubiae has slight hepatotoxicity and nephrotoxicity [92]. Thus, while using these drugs to treat diseases, the same attention should be paid to the evaluation of their potential toxicity, and certain processing or compatibility methods can be used to improve the safety of the clinical medication.

## 6. Discussion

Tibetan medicine is an important part of Chinese traditional medicine and excellent traditional culture, and it is one of the most influential ethnic medicine in China, which possesses a complete theoretical system and has rich experience in the treatment of “jingnisaku disease.” By regulating the balance of the three major factors of rLung, Bad-kan, and mKhris-pa, Tibetan medicine put forward unique treatment methods against the “jingnisaku disease” from the aspects of drugs, diet, and daily life, which has considerable prospects for the treatment of diabetes mellitus and its complication DN. At the same time, we think that the occurrence of “jingnisaku disease” is closely related to the weakening of renal function through the understanding of Tibetan medicine. And modern medicine believes that DN is a secondary kidney disease caused by diabetes mellitus. For the treatment of DN, consideration should be given to renal lesions while treating diabetes. Therefore, the occurrence and development of DN can be effectively prevented and treated by improving renal function and protecting the kidneys.

In view of the knowledge of Tibetan medicine on DN, the search for Tibetan medicines to treat DN will become an idea of new drug development. The results show that there are 60 kinds of drugs for the treatment of kidney diseases in the Tibetan medicine system, and 34 kinds of them have been used in the treatment of DN. These Tibetan medicines are all derived from botanicals and are mainly distributed in 23 families, among which the most frequently used is the leguminous family. It is worth mentioning that *A. kravanh*, *T. chebula*, *M. verticillata*, *R. cordifolia*, *T. terrestris*, *P. longum*, *P. granatum*, and other Tibetan medicines that are most commonly used to treat kidney diseases can also be used in the treatment of DN. Therefore, drugs for the treatment of kidney disease are expected to become a potential source of drugs for the treatment of DN. We find that these drugs mainly exert their effects in the treatment of DN by improving kidney function, lowering blood sugar and blood lipids, and reducing urine protein content, anti-inflammatory, and antioxidative stress and other pathways. However, the mechanisms of some drugs are not yet clear; multidisciplinary approaches should be integrated to perform more pharmacological studies to reveal their mechanisms of action.

To sum up, this study finds that drugs for the treatment of kidney disease can be used as potential drug candidates for the treatment of DN and sorts out the drugs used by Tibetan medicine in the treatment of DN, which expanded the scope of DN medication and provided a new idea for the treatment of DN. In order to make better use of Tibetan medicines, it is necessary to conduct in-depth research of existing Tibetan medicines combined with modern pharmacology, phytochemistry, and other methods to clarify the bioactive ingredients and mechanism of potential drugs for DN and to evaluate their toxic and side effects to improve the effectiveness and safety of Tibetan medicines in the treatment of DN.

## Figures and Tables

**Table 1:** Tibetan medicines for DN in modern research (the order of Tibetan medicine names is from high to low according to the frequency of use).

No.	Latin name	Chinese name	Tibetan name	Family	Medication part	Modern study on the treatment of DN

1	*Amomum kravanh* Pierre ex Gagnep.	Bai Doukou	སུག་སྨེལ་དཀར་པོ།	Zingiberaceae	Fruit	White cardamom volatile oil upregulates the expression of MMP-2, TGF-*β*1, and IGF-2, thereby improving the pathological changes of DN caused by streptozotocin (STZ) and protecting the kidneys of diabetic rats [10].

2	*Terminalia chebula* Retz.	He Zi	རུ་ར	Junzi	Fruit	Chebulinic acid can affect the phosphorylation of VEGF-2 so that VEGF cannot normally play the role of promoting angiogenesis after binding to the receptor, thereby inhibiting the progression of DN. In addition, chebula extract can reduce the blood sugar of diabetic rats caused by STZ and improve the pathological tissue morphology of the kidney in diabetic rats [11].

3	*Malva verticillata* L.	Dong Kui	མ་ནིང་ལྕམ་པ།་	Malvaceae	Fruit	Malva seed is a commonly used drug for the treatment of DN water stasis interaction syndrome [12].

4	*Rubia cordifolia* L.	Qiancao	བཙོད།	Rubiaceae	Root	*R. cordifolia* aqueous root extract exhibited significant antihyperglycemic activities in STZ-induced hyperglycemic rats [13]. Madder mixture can reduce the proteinuria of early DN and is effective in the treatment of early DN [14].

5	*Tribulus terrestris* L.	Ji Li	གཟེ་མ།	Tribulaceae	Fruit	*T. terrestris* has antidiabetic and renal protective capability in alloxan-induced diabetic mice [15]. *T. terrestris* hydroalcoholic extract can reduce the total protein and albumin content of urine in diabetic rats induced by STZ, thereby improving diabetic kidney damage, and has the effect of treating DN [16].

6	*Piper longum* L.	Bi Ba	པི་པི་ལིང་།	Piperaceae	Ear of fruit	Oral administration of *P. longum* dried fruits has shown significant antihyperglycemic, antilipid peroxidative, and antioxidant effects in diabetic rats [17]. The water extract of *P. longum* root has antidiabetic and antihyperlipidemic activities in the STZ-induced diabetes model in rats and also has a protective effect on diabetes-induced kidney damage [18].

7	*Punica granatum* L.	Shiliu	སེ་འབྲུ།	Punicaceae	Fruit	Pomegranate peel tannin can lower the blood sugar of diabetic rats, reduce the oxidation of free radicals on the structure of kidney tissue, and have a beneficial effect on the kidneys of diabetic rats [19]. Pomegranate peel tannin can alleviate the oxidative stress in the renal cortex of diabetic rats, protect the kidneys. and delay the occurrence of DN [20]. Methanolic extract of *P. granatum* leaves has potential antioxidant, antihyperglycemic, and antiglycosylation activities, helping slow down the progression of DN [21].

8	*Berberis kansuensis* Schneid	Xiao Bopi	སྐྱེར་པ།	Berberidaceae	Endothelium	Berberine can improve the damage of vascular endothelial cells, downregulate the expression of VEGF, reduce vascular permeability, protect the function of microvascular endothelial cells, and improve the development of DN [22]. Berberis can upregulate the ornithine content in the serum of DN rats and participate in the metabolism of arginine and proline, thus improving the pathological changes and pharmacodynamic indicators of DN [23].

9	*Mangifera indica* L.	Mang Guohe	ཨ་འབྲས།	Anacardiaceae	Pit	Mangiferin can remarkably ameliorate DN in rats by increasing the activity of glyoxalase 1 [24]. Mangiferin delayed the progression of DN and protected the podocytes by enhancing autophagy under diabetic conditions via the AMPK-mTOR-ULK1 pathway [25]. Mangiferin can reduce the degree of oxidative stress in the kidney and downregulate the expression of CTGF protein in the kidney, delaying kidney damage in diabetic rats [26].

10	*Cinnamomum cassia* Presl	Rou Gui	ཤིང་ཚ།	Lauraceae	Bark	Cinnamon and cinnamon-containing compounds can improve type 2 diabetes and its complications [27]. Cinnamon volatile oil can target to activate E2-related factor 2 (Nrf2), improve metabolic disorders caused by DN, and protect kidney function [28]. The cinnamon extract can reduce diabetic kidney damage by controlling blood sugar, reducing oxidative stress and improving endothelial cell function [29].

11	*Polygonatum sibiricum* Red.	Huang Jing	ར་མཉེ།	Liliaceae	Rhizome	Polygonatum polysaccharide may inhibit the expression of ET-1 and TGF-Pi in diabetic rat models, reduce ECM, delay the occurrence of renal fibrosis, and protect the kidneys of diabetic rats [30]. Polygonatum saponins can inhibit the process of renal tubulointerstitial fibrosis by blocking the activation of the Wnt/*β*-catenin signaling pathway and ultimately play a role in protecting the kidneys of DN rats, which can be used for the prevention and treatment of DN [31, 32].

12	*Eugenia caryophyllata* Thunb	Ding Xiang	ལི་ཤི།	Myrtaceae	Bud	The triterpenoids oleanolic acid (OA) and maslinic acid (MA) in syzygium aromaticum can reduce postprandial hyperglycemia in diabetic rats induced by streptozotocin [33]. OA can enhance the renal function of diabetic rats induced by STZ [34].

13	*Angelica sinensis* (Oliv.) Diels.	Dang Gui	ཏང་ཀུན་ག་པོ།	Umbelliferae	Root	In the treatment of DN, Angelica can reduce urine protein, regulate the expression level of related cytokines, reduce kidney damage, and improve renal function [35]. Angelica polysaccharides can inhibit the excessive proliferation of glomerular mesangium, reduce inflammation, and have a good effect on preventing and treating DN [36]. Angelica polysaccharides can inhibit the differentiation of renal tubular epithelial cells, regulate the production and degradation of extracellular matrix components, and delay the development of diabetic renal fibrosis by reducing the activity of the TGF-*β*1/Smads signaling pathway [37]. Chinese herbal compound containing angelica has certain curative effects in improving renal function and inhibiting the development of DN [38].

14	*Cassia obtusifolia* L.	Jueming	ཐལ་ཀ་རྔོ་རྗེ།	Leguminosae	Seed	Cassia can significantly inhibit the activation of NF-KB and the expression of fibronectin in rats, reduce glomerular hypertrophy, mesangial cell proliferation and extracellular matrix accumulation, and have a significant preventive and therapeutic effect on DN [39]. Cassia anthraquinone glycosides can inhibit the expression of renin and AngII, reduce the content of KIM-1 and *β*2-MG in urine, and have a protective effect on kidney injury in diabetic rats [40].

15	*Cassia tora* L.	Xiao Jueming	ཐལ་ཀ་རྔོ་རྗེ།	Leguminosae	Seed	Same as above

16	*Lycium chinense* Miller	Gouqi	རདྲེ་ཆོར་མ།	Solanaceae	Fruit	Lycium barbarum polysaccharide (LBP) can reduce the production of glycosylation end products in model rats and reduce the secretion of renal IL-8, thus preventing the occurrence of DN [41]. LBP can reduce the expression of MCP1mRNA and ICAM-1mRNA by inhibiting the expression of NF-*κ*B and AngII; it has a significant protective effect on the renal function of DN rabbits and delays the appearance and development of DN [42]. LBP can inhibit the damage of renal tubular epithelial cells in a high glucose environment, inhibit its inflammatory response, and improve the renal function damage of DN rats [43]. LBP can improve the blood sugar level, renal function, and pathological changes of kidney tissue in rats with diabetic kidney injury and has a protective effect on kidney tissue [44].

17	*Lycium barbarum* L.	Ningxia Gouqi	འཕང་མ།	Solanaceae	Fruit	*Lycium barbarum* seed oil can significantly improve kidney function, control the kidney hypertrophy of diabetic mice, and can be used for the treatment of diabetic kidney injury [45].

18	*Astragalus membranaceus* (Fisch.) Bunge.	Huang Qi	བྱི་སྲན་གང་བུ་ཅན་ཀ་།	Leguminosae	Root	Astragaloside may increase the autophagy activity of renal tissue cells by inhibiting PI3K/Akt/FoxO1 signal and slow down the development of type 2 DN [46]. Astragaloside significantly inhibits renal endoplasmic reticulum stress, relieves CHOP-mediated excessive apoptosis of renal tissue cells, significantly reduces proteinuria in DN rats, and improves renal tissue pathological damage in rats [47]. Astragalus can prevent the progression of DN [48].

19	*Trigonella tibetana* (Alef.) Vassiclz.	Hu Luba	ཤུ་མོ་ཟ།	Leguminosae	Seed	Trigonella tibetana can significantly reduce the levels of El, rVIIB2, and blood sugar in diabetic rats; effectively improve DN renal hemodynamics; enhance antioxidant capacity, thereby reducing blood sugar and urine microalbumin; and protect kidney function [49]. *T. tibetana* combined with valsartan in the treatment of patients with DN can significantly reduce their albumin level, which is more effective than valsartan alone, suggesting that *T. tibetana* can be an option for the treatment of DN [50].

20	*Picrorhiza scrophulariiflora* Pennell.	Hu Huanglian	ཧོང་ལེན།	Scrophulariaceae	Rhizome	The total glycosides of rhizoma picrorhizae can significantly improve the hypertrophy of mesangial cells induced by high glucose, reduce the content of intracellular ROS, and increase the level of MMP and reduce Ca^2+^, so as to protect the oxidative stress damage of mesangial cells induced by high glucose [51]. The water extract of rhizoma picrorhizae has a certain curative effect on DN in rats [52, 53].

21	*Rosa laevigata* Michx.	Jin Yingzi	རོང་སལ།	Rosaceae	Fruit	*R. laevigata* extract can improve glucose and lipid metabolism, renal dysfunction, and renal pathological changes in DN rats; delay or prevent the development of DN; and protect the renal function of diabetic rats [54, 55].

22	*Pyrrosia lingua* (Thunb.) Farw.	Shiwei	བྲག་སྤོས་འབྲིང་བ།	Polypodiaceae	Whole grass	*P. lingua* flavonoids can reduce the level of inflammatory factors in the kidney tissue of diabetic rats and effectively improve the kidney injury and inflammatory response of DN [56].

23	*Caesalpinia sappan* L.	Su Mu	མཛོ་མོ་ནིང་།	Leguminosae	Heartwood	Hematoxylon decoction can significantly reduce blood sugar, blood urea nitrogen and creatinine levels in DN rats, reduce kidney damage, and significantly improve kidney function [57]. Hematoxylon can significantly reduce blood CRPI and IL-6 levels in DN rats, reduce urinary protein excretion, improve renal tissue morphology, and may protect the kidneys of early DN rats by inhibiting inflammation [58].

24	*Alpinia oxyphylla* Miq.	Yi Zhi	སུག་སྨེལ་ནག་པོ།	Zingiberaceae	Fruit	*A. oxyphylla* can improve the pathological state of the kidneys and regulate metabolomics and the function of intestinal microbes, so as to achieve the purpose of treating DN [59]. *A. oxyphylla* decoction can effectively lower blood sugar and reduce the excretion of urinary microalbumin, showing good renal protection, and it has a significant effect in the early treatment of DN. It can be used in the clinical treatment of DN [60, 61].

25	*Plantago depressa* Willd.	Che Qiancao	ཐ་རམ།	Plantaginaceae	Whole grass	Plantain water extract can significantly reduce the degree of kidney damage in DN rats, and its mechanism may be related to the inhibition of the p38 MAPK pathway and activation of the PPAR-*γ* pathway [62]. Plantain water extract can significantly reduce kidney damage in DN rats and reduce renal fibrosis [63].

26	*Leontopodium leontopodioides* (Willd.) Beauv.	Huo Rongcao	སྤྲ་ཐོག་པ།	Compositae	Whole grass	Edelweiss quercetin has a significant inhibitory effect on the lipid peroxidation in the kidney tissue of diabetic rats, significantly reduces urinary albumin excretion, improves the glomerular basement membrane and matrix membrane hyperplasia, and can be used as a potential therapeutic drug for DN [64].

27	*Cordyceps sinensis* (Berk.) Sacc.	Dongchong Xiacao	དབྱར་རྩ་དགུན་འབུ།	Ergotaceae	The complex of the subset and the larval carcass	Cordyceps can reduce urine protein and improve renal function in patients with DN [65]. Cordyceps can effectively alleviate renal tubular damage and renal tubular epithelial cell shedding and death, regulate the AMPK/mTOR signaling pathway related to autophagy in renal tubular epithelial cells, and have a protective effect on the kidneys of DN rats [66].

28	*Glycine max* (L.) Merr.	Da Dou		Leguminosae	Seed	Soy isoflavones can protect the kidneys of diabetic rats by improving lipid metabolism disorders [67]. Soy isoflavones have anti-inflammatory and antioxidant effects, which can effectively protect the oxidative stress and inflammation in the kidneys of DN rats [68].

29	*Foeniculum vulgare* (L.) Miller	Hui Xiang	ཟི་ར་དཀར་པོ།	Umbelliflorae	Seed	*F. vulgare* water extract can significantly improve the activity of kidney tissue antioxidant enzymes and the ability to scavenge oxygen free radicals, and reduce the oxidative stress damage of oxygen free radicals to the kidney tissue of diabetic rats, thereby protecting DN [69].

30	*Brassica juncea* (L.)	Jie Zi	ཡུངས་ནག	Cruciferae	Seed	*B. juncea* significantly prevented the rise in creatinine levels; it will delay the development of DN [70].

31	*Sesamum indicum* L.	Hei Zhima	ཏིལ།	Pedaliaceae	Seed	Sesame can improve kidney damage in diabetic rats and increase protein levels [71].

32	*Dolichos lablab* L.	Bian Dou	མོན་སྲན་ལེབ་མོ་དཀར་པོ།	Leguminosae	Seed	Dolichos lablab is a commonly used drug for the treatment of DN water stasis interaction syndrome [12].

33	*Phaseolus radiatus* L.	Cai Dou		Leguminosae	Seed	*P. radiatus* significantly decreased glucose and increased insulin levels, ameliorating the loss of renal function observed in STZ-induced diabetic rats [72].

34	*Rubia membranacea* (Franch.) Diels.	Jin Xiancao	བཙོད།	Rubiaceae	Root	*Rubia membranacea* flavonoids have a certain therapeutic effect on DN [73].
